# Intravascular fasciitis involving the flank of a 21-year-old female: a case report and review of the literature

**DOI:** 10.1186/1756-0500-7-118

**Published:** 2014-02-28

**Authors:** Yan Zheng, Mary George, Frank Chen

**Affiliations:** 1 Department of Pathology, Buffalo General Medical Center, 100 High Street, Buffalo, NY 14203, USA

**Keywords:** Intravascular fasciitis, Flank, Nodular fasciitis

## Abstract

**Background:**

Intravascular fasciitis is an uncommon variant of nodular fasciitis, which is a reactive proliferative lesion of myofibroblasts. Since its identification in 1981, only 32 cases of intravascular fasciitis have been reported in the English literature. The lesion is commonly located in the head, neck, and extremities, with only three cases arising from the trunk. Here we report the fourth case involving the trunk (the flank area).

**Case presentation:**

A 21-year-old African-American female presented with a subcutaneous mass on her flank. Grossly, the mass was red-tan, oval, and well-demarcated, measuring approximately 0.5 cm in diameter. Microscopically, the mass was composed of spindle cells arranged in a swirling and intersecting pattern inside the lumens of two blood vessels. It extended through the vascular walls into the surrounding fibroadipose tissue; in some sections, the spindle cells were intermixed with the perivascular fibrous tissue. Elastin stain revealed remnants of elastic lamina partially surrounding the lesion. The nuclei of the spindle cells were relatively uniform with tapered ends and prominent nucleoli. No significant mitotic activity was observed. Multinucleated giant cells were scattered among the spindle cells, along with infiltrating lymphocytes and extravasated red blood cells. Immunohistochemical stains showed the spindle cells were positive for smooth muscle actin, focally positive for muscle specific actin, and negative for S-100, confirming their myofibroblastic differentiation. The overall morphological and immunohistochemical features are consistent with intravascular fasciitis.

**Conclusion:**

By reporting this rare case, we would like to raise the awareness of this non-neoplastic lesion to avoid misdiagnosing it as a sarcoma with vascular invasion. Previously reported similar cases were also reviewed and compared with this case.

## Background

Intravascular fasciitis is a rare benign lesion characterized by reactive proliferation of myofibroblasts in the superficial or deep fascia with involvement of arteries and/or veins. Intravascular fasciitis is a variant of the more common condition of nodular fasciitis, which does not show vascular invasion. Intravascular fasciitis was originally described by Patchefsky and Enzinger in 1981 [1]. Since then, a total of 32 cases have been reported in the English literature. The lesion is commonly located in the upper extremities, and head and neck, and the upper extremities, with only three cases arising from the trunk [1,2]. Here we report the fourth case involving the trunk area.

## Case presentation

**Figure 1:**
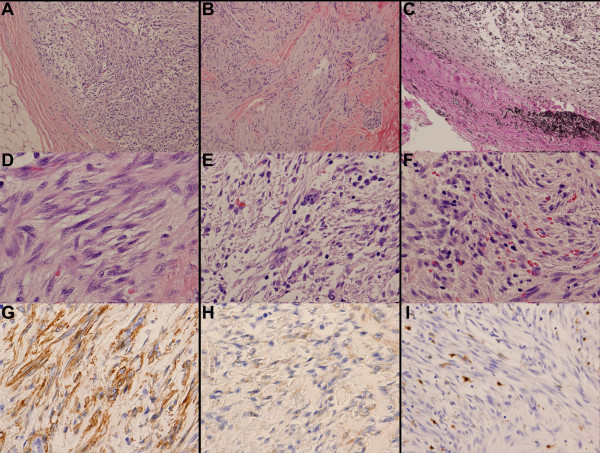
**Intravascular fasciitis. (A)** The mass is composed of spindle cells arranged in an intersecting pattern. **(B)** The mass extends through the vascular walls into the surrounding connective tissue. **(C)** Elastin stain reveals remnants of elastic lamina partially surrounding the lesion. **(D)** The nuclei of the spindle cells are uniform with tapered ends and prominent nucleoli. **(E)** Multinucleated giant cells are scattered within the mass. **(F)** Infiltrating lymphocytes and extravasated red blood cells are present. **(G)** The spindle cells are positive for SMA. **(H)** The spindle cells are focally positive for muscle specific actin. **(I)** The spindle cells are negative for S-100.

The patient was a 21-year-old African-American female who presented with a single nodule on the flank area. Grossly, the subcutaneous nodule was red-tan, oval, and well-demarcated, measuring approximately 0.5 cm in diameter. Microscopically, the mass was composed of spindle cells arranged in a swirling and intersecting pattern inside the lumens of two blood vessels (Figure 1A). It extended through the vascular walls into the surrounding fibroadipose tissue; in some sections, the spindle cells were intermixed with the perivascular fibrous tissue (Figure 1B). Elastin stain revealed remnants of elastic lamina partially surrounding the lesion (Figure 1C). The nuclei of the spindle cells were relatively uniform with tapered ends and prominent nucleoli (Figure 1D). No significant mitotic activity was observed. Multinucleated giant cells were scattered among the spindle cells (Figure 1E), along with infiltrating lymphocytes and extravasated red blood cells (Figure 1F). Immunohistochemical stains showed the spindle cells were positive for smooth muscle actin (SMA; Figure 1G), focally positive for muscle specific actin (Figure 1H), and negative for S-100 (Figure 1I), confirming their myofibroblastic differentiation. The overall morphological and immunohistochemical features are consistent with intravascular fasciitis.

## Discussion

Thirty-two previously reported cases and the current case of intravascular fasciitis are summarized in Table 1[1-14]. Intravascular fasciitis commonly occurs in adolescent and young adult patients with an average age of 24 years old (ranging from 6 months to 66 years old). Of the 33 cases, only six patients were over age of 30. Females and males were equally affected with a female to male ratio close to 1:1. The patients were generally healthy prior to the onset of the lesion. Possible predisposing factors including preceding trauma, thrombosis, and pregnancy-related hormonal changes were noted in a few cases [1,9,10]. The most common locations of the lesion are the upper extremities, head and neck, followed by the lower extremities and trunk. In the majority of cases, the lesion presented as a solitary nodule located subcutaneously or within muscular tissue, although one patient was reported to have a multi-nodular lesion [12]. The size of the lesions ranged from 0.6 to 5 cm in greatest dimension with duration from 2 weeks to 8 years. Other features such as pain/tenderness, mobility, and demarcation of the lesion varied among cases. Intravascular fasciitis is a benign condition, and usually cured by a simple local excision. Of the 16 cases that were followed for 6 months to 20 years, local recurrence was found in three patients [1,7].

**Table 1:** Clinical and pathologic features of the reported cases of intravascular fasciitis

**References**	**#**	**Age (years) and sex**	**Location**	**Gross features**	**Microscopic features**	**IHC features of spindle cells**
Patchefsky et al. 1981 [1]	17	20.5 (range from 0.5 to 57), 8 F and 9 M	Head and neck (n = 5)	1.5 cm^*^ (range from 0.6 to 5), single, firm, non-tender, immobile mass	Feathery, edematous, myxoid, and hyalinized background, giant cells present in 1/3 cases	N/A
			Upper extremity (n = 7)			
			Trunk (n = 2)			
			Lower extremity (n = 3)			
Freedman et al. 1986 [3]	2	19, M	Right posterior mucobuccal fold	2.5 cm, single, firm mass with ulcer	Myxoid and highly vascular background with rare mitotic figures	N/A
		53, M	Left buccal mucosa	2.0 cm, single, firm, immobile mass	Myxoid and locally hyalinized background, no mitotic figures present	N/A
Kahn et al. 1987 [4]	1	20, F	Left lower labial mucosa	1.5 cm, single, firm, immobile mass	Myxoid and highly vascular background, giant cells and mitotic figures present	N/A
Price et al. 1993 [5]	2	17, M	Outer canthus of right eye	2.0 cm, single mass	Myxoid background, giant cells and mitotic figures (Less than 1/HPF) present	N/A
		20, M	Subcutis adjacent to eye and beneath orbicularis oculi	1.0 cm, single, firm, non-tender, mobile mass	Myxoid background, giant cells and mitotic figures (Less than 1/HPF) present	N/A
Samaratunga et al. 1996 [2]	1	49, M	Left inguinal region	3.0 cm, single, firm, non-tender mass	Myxoid background with cleft-like spaces, giant cells and mitotic figures (2/10 HPF) present	(+) Vimentin, SMA
						(-) LMWK, S100
Beer et al. 1996 [6]	1	18, F	Lateral thigh	2.0 cm, single, tender, mobile mass	Focally myxoid and highly vascular background, mitosis present	(-) SMA, S100
Sticha et al. 1997 [7]	1	4, M	Plantar aspect of right foot	3.0 cm, single, firm, tender, immobile mass	Myxomatous and hyalinized background, giant cell and mitosis present	N/A
Ito et al. 1999 [8]	1	26, M	Flexor side of right forearm	Single, tender mass	Fibrous and vascular background, giant cells and mitotic figures (up to 1/10 HPF) present	(+) Vimentin, SMA
						(-) Desmin
Anand et al. 2007 [9]	1	20, F	Hypothenar eminence of right hand	3.0 cm, single, firm, non-tender, mobile mass	Fibrous background, giant cells and mitotic figures present	(+) SMA
						(-) S100, desmin
Sugaya et al. 2007 [10]	1	66, M	Medial border of right foot	0.3 cm, single, non-tender, mobile mass	Myxoid background, no giant cells present, rare mitotic figures	(+) Vimentin
						(-) SMA, cytokeratin, S100, desmin, CD31, CD34, c-kit
Pantanowitz et al. 2008 [11]	1	17, M	Wrist	1.2 cm, single mass	N/A	N/A
Wang et al. 2009 [12]	1	28, F	Left leg	Multiple, firm, non-tender masses	Myxoid background, no giant cells or mitotic figures present	(+) Vimentin, SMA
						(-) Ketatin, S100, desmin
Chi et al. 2012 [13]	1	20, F	Upper lip	0.5 cm, single, firm, non-tender, mobile mass	Giant cells and mitotic figures (11/10 HPF) present	(+) SMA
						(-) S100
Reiser et al. 2012 [14]	1	58, F	Right cheek	1.7 cm, single mass	Focally myxoid and highly vascular background, no giant cells present, rare mitotic figures	(+) SMA
						Focal (+) Bcl-2
						(-) Pankeratin, S100, desmin, EMA
Current case, 2013	1	21, F	Flank	0.5 cm, single mass	Fibrous background, giant cells present with no mitotic figures	(+) SMA
						Focal (+) muscle specific actin
						(-) S100

^*^Size in greatest dimension.

EMA: epithelial membrane antigen.

HPF: high power field.

LMWK: low molecular weight keratin.

N/A: not applicable.

SMA: smooth muscle actin.

Microscopically, intravascular fasciitis was characterized by a spindle cell proliferation inside the lumens or associated with the walls of arteries or veins of all sizes. Depending on the numbers of blood vessels involved and the longitudinal extension of the lesion, the mass exhibited a single or multi-nodular appearance. In two cases, organizing thrombi were found within the lesion [1,10]. One feature that easily mimics a sarcoma is the infiltrating growth. Indeed, it was not uncommon to find that the lesion extended through the vascular walls into the surrounding connective tissue and neighboring blood vessels, but the overlying epidermis was usually intact. In the original report by Patchefsky and Enzinger, they compared the sizes of intravascular and soft tissue components, and concluded that the soft tissue component was dominant; this feature was not reported in the remainder of the cases [1]. The spindle cells were arranged in a storiform pattern or haphazard manner, with plump vesicular nuclei, and in some cases with prominent nucleoli. Mitotic activity ranged from absent to prominent. However, unlike a soft tissue sarcoma, significant cytologic pleomorphism and abnormal mitotic figures were absent. The background stroma varied from a dense hyalinized to edematous, myxoid appearance. Scattered multinucleated giant cells were noted in more than one third of cases including the current one [1,2,4,5,7-9,13]. Lymphocytes and red blood cells were often seen as well.

Immunohistochemistry studies showed that the spindle cells were positive for vimentin and SMA, negative for keratin, S100 protein, desmin, CD31, CD34, and c-kit, confirming their myofibroblastic differentiation. The multinuclear giant cells were CD68 positive, suggesting that they are cells of histiocytic origin.

The pathogenesis of intravascular fasciitis has yet to be understood. The immunohistochemical characteristics of the spindle cells confirm their myofibroblastic origin. However, the factors initiating myofibroblast proliferation have not been clearly identified. Possible risk factors that have been proposed include preceding trauma, thrombosis, and high levels of estrogen. Specifically, two patients developed the lesion following trauma [1,5]. Organizing thrombi were found in two cases [1,10]. The occurrence of intravascular fasciitis in a 16-week pregnant woman, and a previous report showing weak expression of estrogen receptor in nodular fasciitis, led the authors to propose that pregnancy-related estrogen changes might be a predisposing factor [9]. However these conditions were associated with only a minority of cases. More studies are required to clarify their roles in the development of intravascular fasciitis.

Intravascular fasciitis can be misdiagnosed as a sarcoma with vascular invasion because of its intravascular proliferation. However, the cytologic features of intravascular fasciitis, including the absence of large atypical hyperchromatic nuclei and abnormal mitotic figures, are helpful in distinguishing these two conditions [15].

## Conclusions

Intravascular fasciitis is a rare variant of nodular fasciitis associated with blood vessels. By reporting this rare case, we would like to raise the awareness of this non-neoplastic lesion to avoid misdiagnosis.

## Consent

Written informed consent was obtained from the patient for publication of this case report and accompanying images. A copy of the written consent is available for review by the Editor-in-Chief of this journal.

## Abbreviations

SMA: Smooth muscle actin; LMWK: Low molecular weight keratin; EMA: Epithelial membrane antigen.

## Competing interests

The authors declare that they have no competing interests.

## Authors’ contributions

MG collected and interpreted data, and made the diagnosis. YZ and FC were the major contributors in writing and revising the manuscript. All authors read and approved the final manuscript.
